# 3,5,6-Tri­chloro­pyridin-2-ol

**DOI:** 10.1107/S241431462401126X

**Published:** 2024-11-22

**Authors:** Tashonda M. Vaughn, Saneei Soheil, Olalekan M. Ogundele, Frank R. Fronczek, Rao M. Uppu

**Affiliations:** a Department of Environmental Toxicology Southern University and A&M College Baton Rouge Louisiana 70813 USA; b Department of Comparative Biomedical Sciences School of Veterinary Medicine Louisiana State University,Baton Rouge Louisiana 70810 USA; c Department of Chemistry Louisiana State University,Baton Rouge LA 70803 USA; University of Aberdeen, United Kingdom

**Keywords:** crystal structure, chlorpyrifos, triclopyr derivatives

## Abstract

The title compound, C_5_H_2_Cl_3_NO, is almost planar. In the crystal, the mol­ecules form centrosymmetric hydrogen-bonded dimers through pairwise O—H⋯N inter­actions to generate *R*^2^_2_(8) loops.

## Abstract 2

The title compound is almost planar. In the crystal, the mol­ecules form centrosymmetric hydrogen-bonded dimers through pairwise O—H⋯N inter­actions to generate *R*^2^_2_(8) loops.

## Graphical abstract

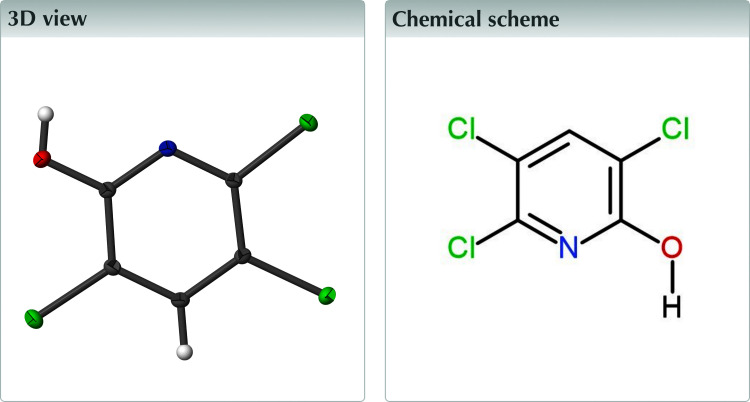

## Structure description

3,5,6-Tri­chloro-2-pyridinol (TCP, C_5_H_2_Cl_3_NO) is the primary degradation product of chlorpyrifos (CPP, C_9_H_11_Cl_3_NO_3_PS) and chlorpyrifos-methyl (CPFM, C_7_H_7_Cl_3_NO_3_PS), two of the most widely used organophosphate insecticides in agriculture (Bouchard *et al.*, 2011 ▸). TCP has been shown to intensify the toxic effects of CPF(*M*), leading to endocrine disruption, cellular toxicity, and organ damage (Gao *et al.*, 2021 ▸; Li *et al.*, 2020 ▸). It enhances the impact of CPF(*M*) on testosterone synthesis and Sertoli cell function by inhibiting testosterone binding to androgen receptors, furthering hormonal disruption through pathways involving luteinizing hormone and signaling mol­ecules such as CREB and Star, essential for testosterone production. TCP also downregulates genes critical for spermatogenesis, posing potential risks to male fertility (Mansukhani *et al.*, 2024 ▸). Mol­ecular modeling indicates that TCP inter­acts with sex-hormone-binding globulin, potentially aggravating hormonal imbalances (Haza­rika *et al.*, 2019 ▸).

Beyond endocrine effects, TCP exhibits direct cytotoxicity (Gao *et al.*, 2021 ▸) and may bind to DNA in a groove-binding manner similar to Hoechst, possibly favoring specific base-pair regions without significantly distorting the DNA structure (Bailly *et al.*, 1993 ▸; Bucevičius *et al.*, 2018 ▸; Kashanian *et al.*, 2012 ▸). Studies have demonstrated substantial cellular damage in human embryonic kidney cells (HEK 293) following TCP exposure, signaling a risk of kidney toxicity (Van Emon *et al.*, 2018 ▸). TCP is further linked to hepatotoxicity and nephrotoxicity in animal models, where it accumulates in vital organs and may cause structural and functional damage (Deng *et al.*, 2016 ▸). Additionally, age-dependent sensitivity to TCP has been observed, with pre-weanling rats displaying heightened vulnerability due to pharmacokinetic differences (Timchalk *et al.*, 2002 ▸).

TCP is notable for its long half-life in soil, ranging from 65 to 360 days depending on environmental conditions, and its high solubility in water (80.9 mg l^−1^), facilitating contamination of surface and groundwater (Zhao *et al.*, 2017 ▸; Timchalk *et al.*, 2002 ▸). This persistence raises substantial concerns about bioaccumulation, biomagnification, and ecosystem disruption, especially in aqua­tic environments where TCP has been found to be toxic to organisms (Echeverri-Jaramillo *et al.*, 2020 ▸; Van Emon *et al.*, 2018 ▸). TCP levels in human urine serve as biomarkers for CPF(*M*) exposure, aiding in occupational and environmental exposure assessments (Bouchard *et al.*, 2011 ▸).

In the United States, the EPA revoked all food-related uses of CPF(*M*) in 2021, effectively banning its use on crops intended for human consumption; however, a November 2023 court ruling temporarily reinstated CPF(*M*) tolerances while the EPA reconsiders its decision (EPA, 2023 ▸). In the European Union, both CPF and CPFM were banned in 2020, with strict limits on residue levels in food (EFSA, 2020 ▸). As of 2024, CPF(*M*) remains restricted for non-food uses in some areas, with existing stocks allowed under controlled conditions, though further bans and stricter regulations are anti­cipated. While these restrictions are in place, it is important to note that TCP can also result from the soil and microbial degradation of triclopyr, triclopyr but­oxy­ethyl ester and triclopyr tri­ethyl­amine salt, three commonly used pyridine-based herbicides for managing woody plants, vines, and broadleaf weeds (Cessna *et al.*, 2002 ▸; Deng *et al.*, 2016 ▸; Dias *et al.*, 2017 ▸). The primary concern with these herbicides is non-target toxicity.

**Figure 1:**
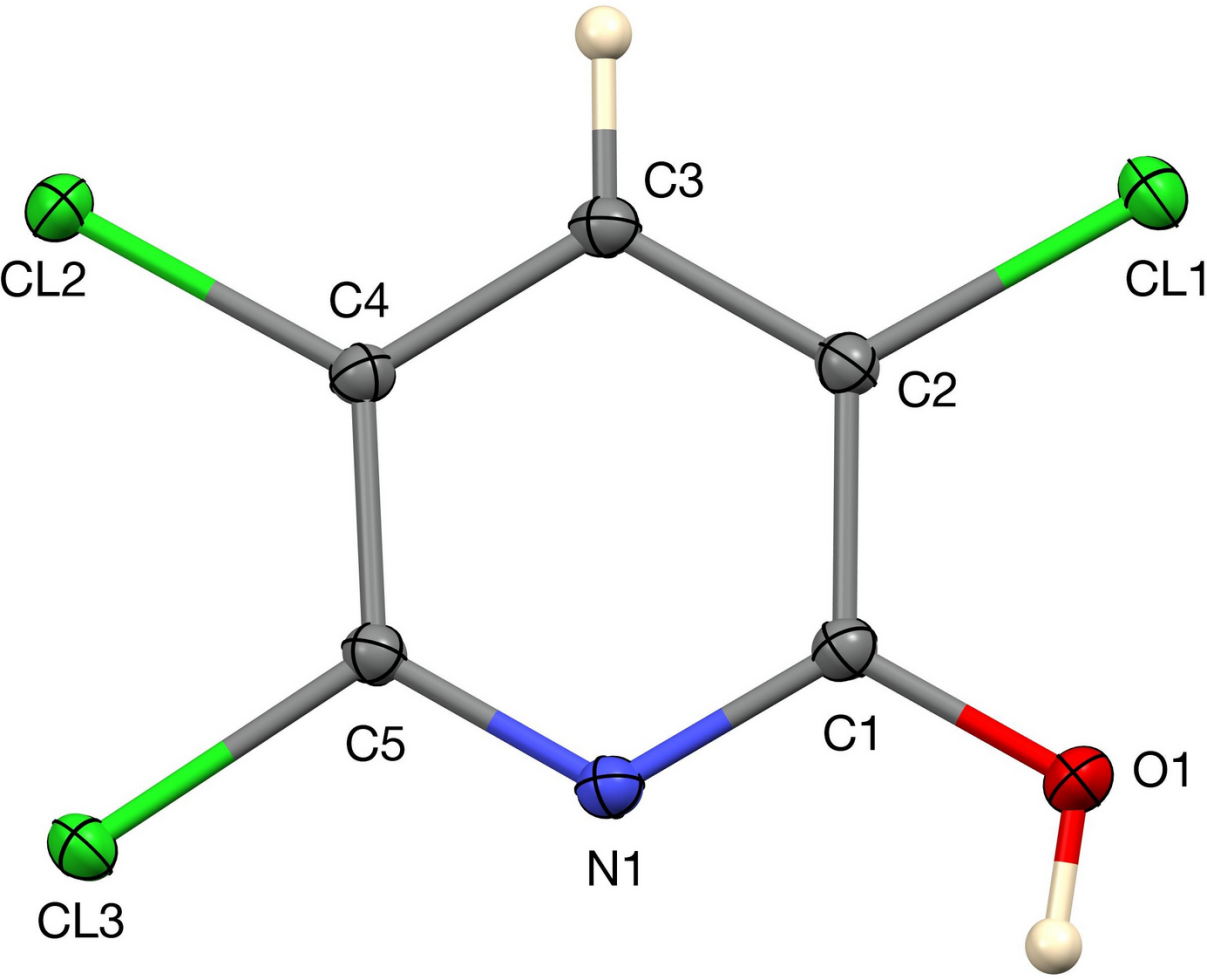
The asymmetric unit of TCP with 50% displacement ellipsoids.

**Figure 2:**
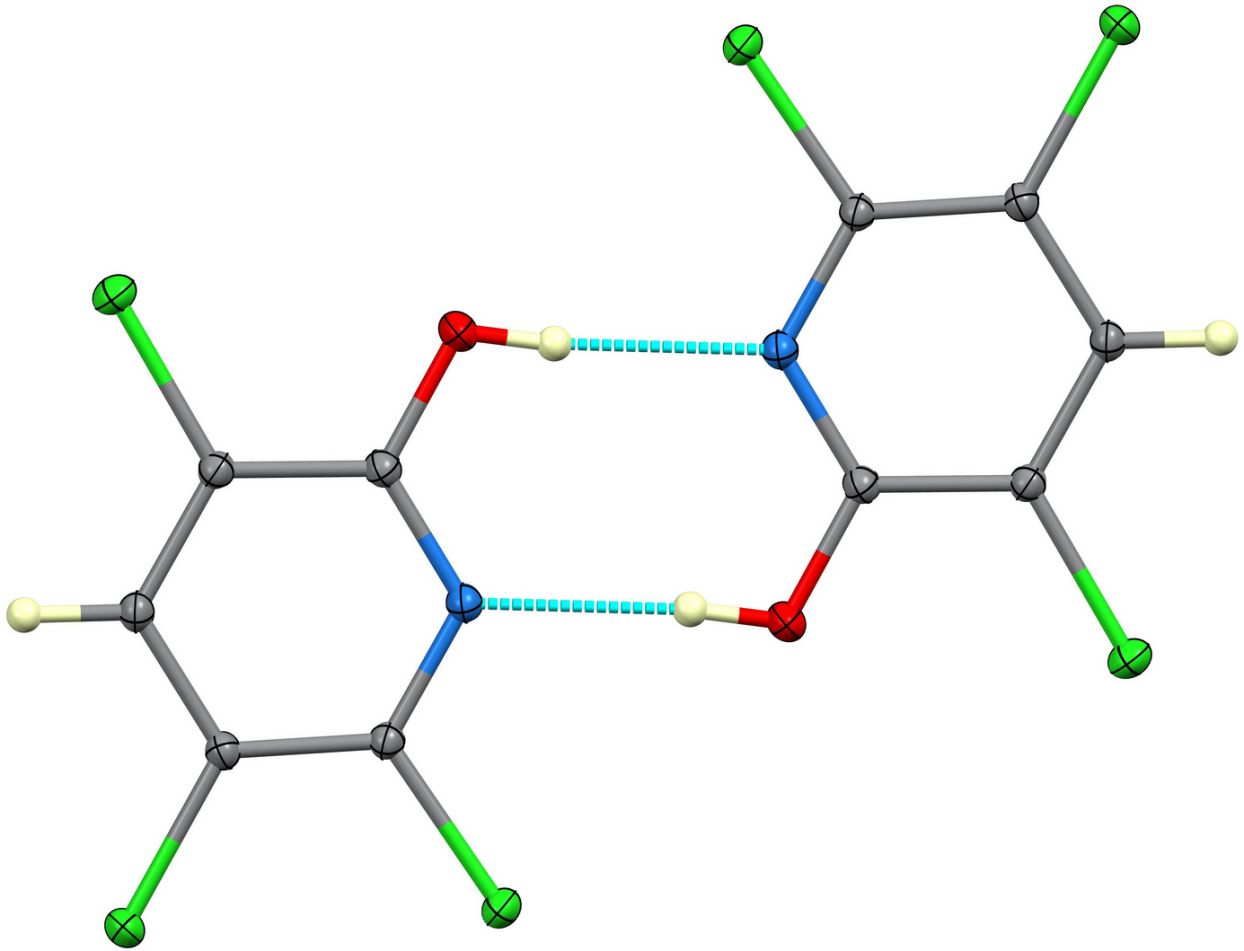
The centrosymmetric hydrogen-bonded dimer.

**Figure 3:**
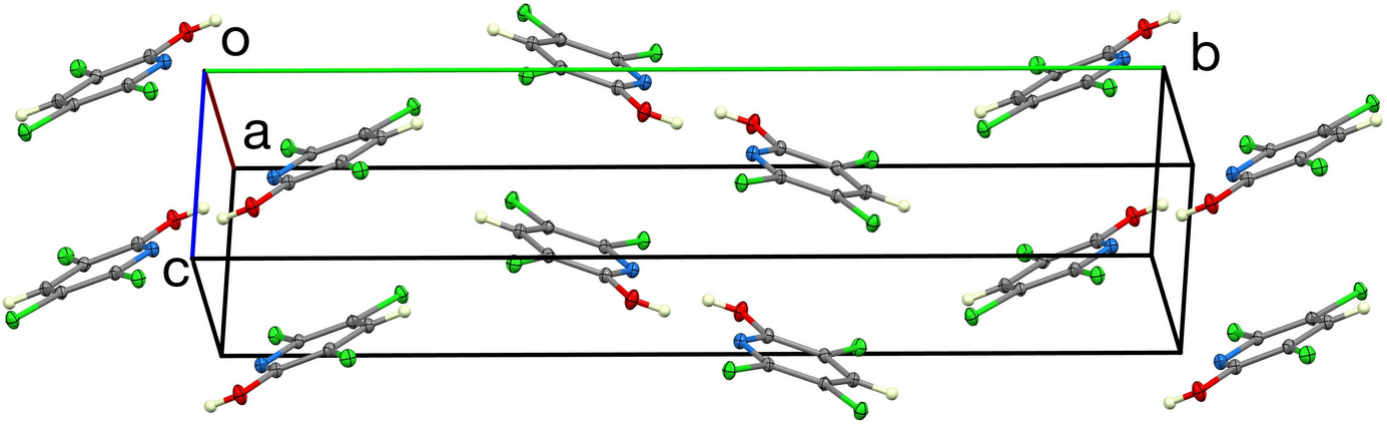
The unit-cell packing.

Given its widespread relevance to human and animal health, and to support the identification of potential mol­ecular targets in biological systems, we have investigated the crystal structure of TCP: it crystallizes in the monoclinc space group *P*2_1_/*c* with one mol­ecule in the asymmetric unit (Fig. 1 ▸). The mol­ecule is close to planar, with the six atoms of the pyridine ring lying a mean of 0.007 Å from their best plane. The Cl atoms lie out of this plane by an average of 0.058 Å, and the O atom lies 0.0430 (14) Å out of plane. The C—N distances are 1.3343 (11) and 1.3347 (11) Å, the C—Cl distances fall in the range 1.7189 (8)–1.7202 (8) Å and the C—O distance is 1.3207 (11) Å. In the crystal, the mol­ecules form centrosymmetric hydrogen-bonded dimers through pairwise O—H⋯N inter­actions (Table 1 ▸) to generate 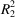
(8) loops. No other directional inter­actions could be identified. The hydrogen-bonded dimer is shown in Fig. 2 ▸, and the unit cell is illustrated in Fig. 3 ▸.

## Synthesis and crystallization

3,5,6-Tri­chloro-2-pyridinol, C_5_H_2_Cl_3_NO (CAS 6515–38-4) was obtained from AmBeed (Arlington Heights, Illinios, USA) and was used without further purification. Crystals in the form of colorless laths were prepared by slow cooling of a nearly saturated solution of the title compound in boiling deionized water (resistance *ca*. 18 MΏ cm^−1^).

## Refinement

Crystal data, data collection and structure refinement details are summarized in Table 2 ▸.

## Supplementary Material

Crystal structure: contains datablock(s) I. DOI: 10.1107/S241431462401126X/hb4494sup1.cif

Structure factors: contains datablock(s) I. DOI: 10.1107/S241431462401126X/hb4494Isup2.hkl

Supporting information file. DOI: 10.1107/S241431462401126X/hb4494Isup3.cml

CCDC reference: 2403937

Additional supporting information:  crystallographic information; 3D view; checkCIF report

## Figures and Tables

**Table 1:** Hydrogen-bond geometry (Å, °)

*D*—H⋯*A*	*D*—H	H⋯*A*	*D*⋯*A*	*D*—H⋯*A*
O1—H1⋯N1^i^	0.821 (19)	1.919 (19)	2.7371 (10)	174.3 (19)

Symmetry code: (i) 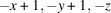.

**Table 2:** Experimental details

Crystal data	
Chemical formula	C_5_H_2_Cl_3_NO
*M*_r_	198.43
Crystal system, space group	Monoclinic, *P*2_1_/*c*
Temperature (K)	100
*a*, *b*, *c* (Å)	6.1616 (3), 22.3074 (10), 5.0396 (2)
β (°)	99.356 (1)
*V* (Å^3^)	683.47 (5)
*Z*	4
Radiation type	Ag *K*α, λ = 0.56086 Å
μ (mm^−1^)	0.64
Crystal size (mm)	0.47 × 0.23 × 0.14

Data collection	
Diffractometer	Bruker D8 Venture DUO with Photon III C14
Absorption correction	Multi-scan (*SADABS*; Krause *et al.*, 2015 ▸)
*T*_min_, *T*_max_	0.840, 0.916
No. of measured, independent and observed [*I* > 2σ(*I*)] reflections	56728, 4816, 4626
*R*_int_	0.035
(sin θ/λ)_max_ (Å^−1^)	0.944

Refinement	
*R*[*F*^2^ > 2σ(*F*^2^)], *wR*(*F*^2^), *S*	0.034, 0.073, 1.33
No. of reflections	4816
No. of parameters	94
H-atom treatment	H atoms treated by a mixture of independent and constrained refinement
Δρ_max_, Δρ_min_ (e Å^−3^)	0.78, −0.50

Computer programs: *APEX4* and *SAINT* (Bruker, 2016 ▸), *SHELXT2018/2* (Sheldrick, 2015 ▸a), *SHELXL2019/1* (Sheldrick, 2015 ▸b), *Mercury* (Macrae *et al.*, 2020 ▸) and *publCIF* (Westrip, 2010 ▸).

## full crystallographic data

### 3,5,6-Trichloropyridin-2-ol Crystal data

**Table:**

C_5_H_2_Cl_3_NO	*F*(000) = 392
*M**_r_* = 198.43	*D*_x_ = 1.928 Mg m^−^^3^
Monoclinic, *P*2_1_/*c*	Ag *K*α radiation, λ = 0.56086 Å
*a* = 6.1616 (3) Å	Cell parameters from 9745 reflections
*b* = 22.3074 (10) Å	θ = 2.6–31.9°
*c* = 5.0396 (2) Å	µ = 0.64 mm^−^^1^
β = 99.356 (1)°	*T* = 100 K
*V* = 683.47 (5) Å^3^	Lath fragment, colourless
*Z* = 4	0.47 × 0.23 × 0.14 mm

### 3,5,6-Trichloropyridin-2-ol Data collection

**Table:**

Bruker D8 Venture DUO with Photon III C14 diffractometer	4626 reflections with *I* > 2σ(*I*)
Radiation source: IµS 3.0 microfocus	*R*_int_ = 0.035
φ and ω scans	θ_max_ = 32.0°, θ_min_ = 2.6°
Absorption correction: multi-scan (SADABS; Krause *et al.*, 2015)	*h* = −11→11
*T*_min_ = 0.840, *T*_max_ = 0.916	*k* = −42→42
56728 measured reflections	*l* = −9→9
4816 independent reflections	

### 3,5,6-Trichloropyridin-2-ol Refinement

**Table:**

Refinement on *F*^2^	Primary atom site location: dual
Least-squares matrix: full	Hydrogen site location: mixed
*R*[*F*^2^ > 2σ(*F*^2^)] = 0.034	H atoms treated by a mixture of independent and constrained refinement
*wR*(*F*^2^) = 0.073	*w* = 1/[σ^2^(*F*_o_^2^) + (0.0157*P*)^2^ + 0.4001*P*] where *P* = (*F*_o_^2^ + 2*F*_c_^2^)/3
*S* = 1.33	(Δ/σ)_max_ = 0.001
4816 reflections	Δρ_max_ = 0.78 e Å^−^^3^
94 parameters	Δρ_min_ = −0.50 e Å^−^^3^
0 restraints	

### 3,5,6-Trichloropyridin-2-ol Special details

**Table:**

Geometry. All esds (except the esd in the dihedral angle between two l.s. planes) are estimated using the full covariance matrix. The cell esds are taken into account individually in the estimation of esds in distances, angles and torsion angles; correlations between esds in cell parameters are only used when they are defined by crystal symmetry. An approximate (isotropic) treatment of cell esds is used for estimating esds involving l.s. planes.
Refinement. Both H atoms were located in difference maps and the one on C was treated as riding in a geometrically idealized position with C—H distance = 0.95 Å, while the coordinates for the one on O were refined. Hydrogen displacement parameters were assigned as *U*_iso_(H) = 1.2*U*_eq_ for the attached C atom and 1.5*U*_eq_ for the attached O atom.

### 3,5,6-Trichloropyridin-2-ol Fractional atomic coordinates and isotropic or equivalent isotropic displacement parameters (Å^2^)

**Table:**

	*x*	*y*	*z*	*U*_iso_*/*U*_eq_	
Cl1	0.93128 (4)	0.66668 (2)	0.00185 (5)	0.01441 (4)	
Cl2	0.32839 (4)	0.69555 (2)	0.65348 (4)	0.01403 (4)	
Cl3	0.17530 (4)	0.56134 (2)	0.51565 (5)	0.01375 (4)	
O1	0.72509 (13)	0.54889 (3)	−0.08395 (16)	0.01555 (12)	
H1	0.661 (3)	0.5167 (9)	−0.111 (4)	0.023*	
N1	0.47368 (12)	0.55968 (3)	0.20415 (15)	0.01124 (10)	
C1	0.63850 (14)	0.58150 (4)	0.09104 (17)	0.01105 (11)	
C2	0.72170 (14)	0.63936 (4)	0.15463 (17)	0.01084 (11)	
C3	0.62942 (14)	0.67423 (4)	0.33201 (17)	0.01121 (12)	
H3	0.684573	0.713275	0.378054	0.013*	
C4	0.45373 (14)	0.65136 (4)	0.44307 (17)	0.01050 (11)	
C5	0.38383 (13)	0.59348 (4)	0.37596 (17)	0.01031 (11)	

### 3,5,6-Trichloropyridin-2-ol Atomic displacement parameters (Å^2^)

**Table:**

	*U*^11^	*U*^22^	*U*^33^	*U*^12^	*U*^13^	*U*^23^
Cl1	0.01297 (8)	0.01471 (8)	0.01651 (9)	−0.00232 (6)	0.00528 (6)	0.00075 (6)
Cl2	0.01719 (9)	0.01155 (7)	0.01464 (8)	−0.00103 (6)	0.00649 (6)	−0.00281 (6)
Cl3	0.01369 (8)	0.01177 (7)	0.01685 (8)	−0.00229 (6)	0.00571 (6)	0.00032 (6)
O1	0.0175 (3)	0.0118 (2)	0.0191 (3)	−0.0019 (2)	0.0083 (2)	−0.0045 (2)
N1	0.0115 (2)	0.0093 (2)	0.0132 (3)	−0.00046 (19)	0.0027 (2)	−0.0007 (2)
C1	0.0112 (3)	0.0095 (3)	0.0126 (3)	0.0002 (2)	0.0025 (2)	−0.0006 (2)
C2	0.0105 (3)	0.0103 (3)	0.0119 (3)	−0.0008 (2)	0.0023 (2)	0.0005 (2)
C3	0.0124 (3)	0.0090 (3)	0.0121 (3)	−0.0011 (2)	0.0017 (2)	−0.0003 (2)
C4	0.0116 (3)	0.0090 (3)	0.0110 (3)	0.0002 (2)	0.0022 (2)	−0.0006 (2)
C5	0.0103 (3)	0.0090 (3)	0.0117 (3)	−0.0005 (2)	0.0021 (2)	0.0002 (2)

### 3,5,6-Trichloropyridin-2-ol Geometric parameters (Å, º)

**Table:**

Cl1—C2	1.7191 (8)	N1—C1	1.3347 (11)
Cl2—C4	1.7202 (8)	C1—C2	1.4063 (12)
Cl3—C5	1.7189 (8)	C2—C3	1.3757 (12)
O1—C1	1.3207 (11)	C3—C4	1.3937 (12)
O1—H1	0.821 (19)	C3—H3	0.9500
N1—C5	1.3343 (11)	C4—C5	1.3854 (11)
			
C1—O1—H1	110.9 (13)	C2—C3—H3	120.5
C5—N1—C1	119.73 (7)	C4—C3—H3	120.5
O1—C1—N1	120.16 (8)	C5—C4—C3	118.30 (7)
O1—C1—C2	119.06 (8)	C5—C4—Cl2	122.12 (6)
N1—C1—C2	120.78 (8)	C3—C4—Cl2	119.58 (6)
C3—C2—C1	119.55 (8)	N1—C5—C4	122.66 (8)
C3—C2—Cl1	120.69 (6)	N1—C5—Cl3	116.53 (6)
C1—C2—Cl1	119.73 (6)	C4—C5—Cl3	120.80 (6)
C2—C3—C4	118.95 (7)		
			
C5—N1—C1—O1	−178.44 (8)	C2—C3—C4—C5	1.98 (12)
C5—N1—C1—C2	1.22 (13)	C2—C3—C4—Cl2	−177.11 (7)
O1—C1—C2—C3	178.53 (8)	C1—N1—C5—C4	0.35 (13)
N1—C1—C2—C3	−1.14 (13)	C1—N1—C5—Cl3	−179.07 (7)
O1—C1—C2—Cl1	0.66 (12)	C3—C4—C5—N1	−1.97 (13)
N1—C1—C2—Cl1	−179.01 (7)	Cl2—C4—C5—N1	177.10 (7)
C1—C2—C3—C4	−0.50 (13)	C3—C4—C5—Cl3	177.43 (6)
Cl1—C2—C3—C4	177.35 (7)	Cl2—C4—C5—Cl3	−3.51 (11)

### 3,5,6-Trichloropyridin-2-ol Hydrogen-bond geometry (Å, º)

**Table:**

*D*—H···*A*	*D*—H	H···*A*	*D*···*A*	*D*—H···*A*
O1—H1···N1^i^	0.821 (19)	1.919 (19)	2.7371 (10)	174.3 (19)

Symmetry code: (i) −*x*+1, −*y*+1, −*z*.
